# Isolation and Identification of Organics-Degrading Bacteria From Gas-to-Liquid Process Water

**DOI:** 10.3389/fbioe.2020.603305

**Published:** 2021-01-15

**Authors:** Riham Surkatti, Zulfa A. Al Disi, Muftah H. El-Naas, Nabil Zouari, Mark C. M. Van Loosdrecht, Udeogu Onwusogh

**Affiliations:** ^1^Gas Processing Center, Qatar University, Doha, Qatar; ^2^Department of Biotechnology, Delft University of Technology, Delft, Netherlands; ^3^Department of Biological & Environmental Sciences, College of Arts Sciences, Qatar University, Doha, Qatar; ^4^Qatar Shell Research and Technology Center, Doha, Qatar

**Keywords:** GTL process water, isolation, identification, biodegradation, COD reduction

## Abstract

The gas-to-liquid (GTL) process generates considerable amounts of wastewater that are highly acidic and characterized by its high chemical oxygen demand (COD) content, due to the presence of several organic pollutants, such as alcohols, ketones, aldehydes, and fatty acids. The presence of these organics in the process water may lead to adverse effect on the environment and aquatic life. Thus, it is necessary to reduce the COD content of GTL process water to an acceptable limit before discharging or reusing the treated water. Due to several advantages, biological treatment is often utilized as the main step in GTL process water treatment plants. In order to have a successful biotreatment process, it is required to choose effective and suitable bacterial strains that have the ability to degrade the organic pollutants in GTL process water. In this work, bacterial strains were isolated from the GTL process water, identified by 16S rRNA gene sequencing and then used in the biodegradation process. The detailed identification of the strains confirmed the presence of three organics-degrading bacteria identified as *Alcaligenes faecalis, Stenotrophomonas* sp., and *Ochrobactrum* sp. Furthermore, biodegradation experiments were carried out and confirmed that the pure culture as well as the mixed culture consortium of the bacterial strains has the ability to reduce the organic pollutants in GTL process water. However, the growth rate and biodegradation efficiency depend on the type of strains and the initial COD content. Indeed, the removal percentage and growth rate were enhanced after 7 days for all cultures and resulted in COD reduction up to 60%. Moreover, the mixed culture of bacterial strains can tolerate and treat GTL process water with a variety of ranges of COD contents.

## Introduction

Qatar is the capital of natural gas production and hosts the largest gas-to-liquid (GTL) plant in the world. During the GTL process, considerable amounts of water are often generated, due to the chemistry nature of the Fischer–Tropsch (F-T) process that is the main process in natural gas plants. The generated GTL process water contains several dissolved hydrocarbons that cannot be directly used or discharged to the aquatic environment. GTL process water is characterized by high acidity and high chemical oxygen demand (COD) content that can reach 32,000 mg/L. The water is mainly contaminated with non-acid oxygenated (NAO) hydrocarbons, including ketones, aldehydes, alcohols, ethers, and esters (Zacharia et al., 2018). Thus, an appropriate treatment process should be applied to reduce the concentration of organics in GTL process water to the acceptable discharge limit. Since F-T water contains volatile organics and light oxygenates such as carbonyl compounds and C_1_-C_3_ alcohols which have boiling points less than that of water, they are typically treated using distillation or stripping columns. Therefore, the GTL wastewater treatment plant usually requires a pretreatment step such as stripping or distillation column to reduce the content of COD before the biological treatment process (Surkatti et al., 2020). The wastewater generated from the stripping/distillation column still contains large quantities of carboxylic acids and other oxygenates, which need to be treated biologically (Enyi et al., 2013).

The conventional activated sludge process, constructed wetlands, and trickling filters are nowadays among the most common biological technologies for the treatment of wastewaters, in addition to different types of membrane bioreactors (Shokrollahzadeh et al., 2008). For GTL process water, several biological treatments were combined with other treatment processes to achieve high COD reduction efficiency (Wang et al., 2016). However, the use of traditional anaerobic suspended sludge process has a major limitation in wastewater treatment. Therefore, new biological reactors were developed for the treatment of industrial wastewater, in which bacterial strains were used in the form of free or immobilized systems (El-Naas et al., 2013, 2016; Bouabidi et al., 2019).

In biological treatments, chemo-organotrophic species are considered as the main microbial degraders of organic pollutants in contaminated wastewaters (Fritsche and Hofrichter, 2008). A large range of organic compounds can be used by these chemoheterophic microorganisms, especially bacteria, as carbon and energy sources (Elbeshbishy, 2014). However, searching to extend the range of the organics that can be used, their admissible concentrations, and the efficiency of their removal is leading to continuous efforts deployed to isolate new strains or species having the ability to degrade all organics present in GTL process water. This is more needed for special situations of water generated in arid zones, like the Gulf area, characterized by harsh conditions (Disi et al., 2017). Indeed, the selection of any bacterial strain for a bioremediation process is based on its ability to tolerate harsh conditions, exhibiting a suitable activity for the existing organics in the treated water (Azubuike et al., 2016). Arid and semiarid areas are characterized by harsh weather with high temperatures that can affect bacterial populations and create a dynamic diversity of these bacteria, based on their ability to adapt by acquiring new metabolic activities and suitable surfactant production (Elazzazy et al., 2015). Local bacteria are then needed for the treatment of local GTL process water. Moreover, under harsh conditions, indigenous microorganisms have adapted to develop a specific metabolism, effective for these weathered organics (Kumar and Gopal, 2015). Indeed, many failures of bioremediation applications in areas characterized by harsh weather and soils can be attributed to the use of unacclimated bacteria and their associated activities (Disi et al., 2017).

The novelty of this work resides in the collection, isolation, and identification of indigenous bacteria from GTL process water available in Qatar. The same bacterial strains are then utilized for the biodegradation of major organic contaminants in local GTL process water. The main approach employed in this work is multidisciplinary, combining environmental microbiology, biochemistry, and bioremediation. This is important from fundamental and applied points of view for specific GTL process water generated and treated at harsh conditions.

## Materials and Methods

### Industrial Water Samples

Process water samples were collected from a local GTL plant in Qatar, in which two types of water samples were analyzed and referred to as pretreated GTL process water and original GTL process water. The physical and chemical characteristics of the GTL process water samples are shown in Table 1. GTL process water is characterized by its high acidity and organic contents (high COD).

**Table 1:** Physical and chemical characteristics of GTL.

**Characteristic**	**GTL process water**	**Pretreated GTL process water**
COD (mg/L)	5,000–7,000	1,800–2,000
TOC (mg/L)	1,500–1,700	700–800
pH	2.9	2.9
Conductivity	0.435	0.430

In order to investigate the organics present in GTL process water, a qualitative analysis using GC-MS was carried out for the GTL process water (6,000 mg/L COD) and for pretreated GTL process water. In general, GTL process water contains alcohols, ketones, volatile fatty acids, ester, and other aliphatics. Based on the analysis, the main organic contaminants are short-chain alcohols and long-chain alcohols; it also contains some fatty acids, such as propenoic acid, butanoic acid, and acetoacetic acid, in addition to the esters, aliphatics, and ketones such as methyl ketone and pentanone. However, the type of organic pollutants is varied for the raw GTL process water and the pretreated GTL process water since the pretreated wastewater contains alcohols, ketones, and some aliphatics.

### Isolation and Purification of Bacterial Strains From Enrichment Cultures

**Figure 1:**
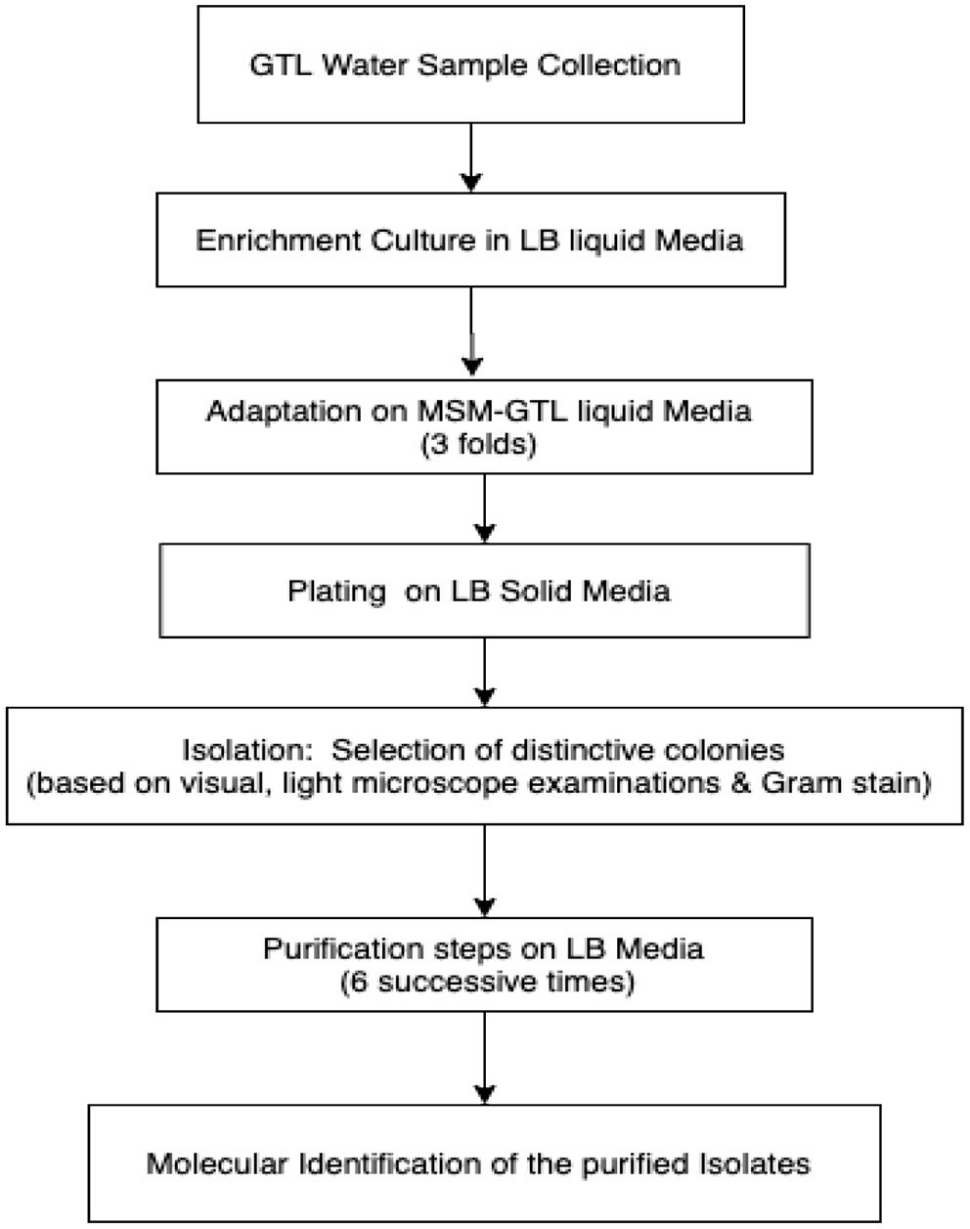
Flowchart illustrating the isolation strategy.

Luria–Bertani (LB) medium was used for the enrichment culture, isolation, and purification of the bacterial strains (Figure 1).

One milliliter of the GTL process water was suspended in 20 ml liquid LB medium as the first enrichment step. The liquid cultures were incubated at 30°C in a rotating shaker set at 200 rpm for 3 days. At the end of the incubation period, 2 ml from each liquid culture was transferred to 20 ml mineral salts medium (MSM)-GTL as the adaptation step. The adaptation steps were repeated three times. Then, aliquots (100 μl) of the MSM-GTL enrichment cultures were spread on solid LB agar medium. The LB plates were incubated at 30°C for 24 h. Isolates exhibiting distinct colonial morphologies were isolated and transferred to separate LB agar plates for further purification. Consequent purification of the bacterial isolates was repeated six times using the streak plate method until pure isolates were obtained (Survery et al., 2005). Stock bacterial cultures were preserved at −80°C in 30% glycerol until use.

### Evaluation of the Potential of Bacterial Strains to Remove COD in GTL Process Water

Biodegradation experiments were performed using MSM liquid medium containing per liter (pH 7.2): NH_4_NO_3_, 4.0 g; Na_2_HPO_4_, 2.0 g; KH_2_PO_4_, 0.53 g; K_2_SO_4_, 0.17 g; MgSO_4_·7H_2_O, 0.10 g; and 1 ml/L of trace element solution (per 100 ml): EDTA, 0.1 g; ZnSO_4_, 0.042; MnSO_4_, 0.178 g; H_3_BO_3_, 0.05; and NiCl_2_, 0.1 g. The media were prepared using different concentrations of GTL process water. All media were sterilized by autoclaving at 121°C for 20 min. Analysis was performed after the incubation periods to investigate the reduction in organic pollutants present in GTL wastewater. The bacterial growth was evaluated using the Lambda 25 UV/VIS spectrophotometer at 600 nm. Samples were collected and analyzed at several time intervals to confirm the growth and the biodegradation efficiency of the isolated bacteria. All biodegradation experiments were carried out at optimal conditions, temperature of 30°C and pH 7, since there was no growth or organic removal observed at room temperature (T = 25°C) and pH values (pH 3.0 and 5.0).

### COD Determination

The COD analysis was carried out using a HAC-UV spectrophotometer with COD reagents. The analysis was obtained by adding 2 ml of the water sample into the HAC LCK514 cuvettes and heating for 2 h to complete the reaction between the reagent and water sample. The sample was then transferred to the HAC 3900 to read the COD content in milligrams per liter. Each sample was analyzed in duplicate.

### Molecular Identification of Isolates

The DNA was obtained from cells grown on LB solid media overnight at 30°C. Pure colonies were suspended into Eppendorf tubes filled with 0.5 ml of sterile distilled water. The Eppendorf tubes were incubated in a water bath set at 100°C for 10 min and then placed in an ice bath for 1 min. After centrifugation for 60 s at 12,300 rpm, the supernatant containing total DNA was carefully transferred to a new sterile Eppendorf tube. The amplification of the 16S rRNA gene fragments of ~1.5 kb was carreid out using two universal primers: RibS73sp 5′-AGAGTTTGATCCTGGCTCA-3′ and RibS74sp 5′-AAGGAGGTGATCCAGCCGCA-3′ (Lane, 1991). The PCR reactions were performed in a total volume of 25 μl including MgCl_2_ 1.5 μM, dNTP 0.8 μM, forward primer 1.35 μM, reverse primer 1.35 μM, and 0.5 IU Taq DNA polymerase; 2 μl of genomic DNA from the isolates served as template for the PCR reactions.

The thermocycler program for each PCR reaction was intiated with a 3-min denaturation step set at 94°C, followed by 35 cycles of denaturation steps at 94°C with 45 s each, annealing step at 50°C for 45 s, elongation step at 72°C for 45 s, and then one final 2-min extension step at 72°C. Consequently, the purification of PCR products was acomplished using an Invitrogen PureLink PCR Purification Kit. The DNA sequencing was performed using a Genetic Analyzer-Applied Biosystems 3,500 Series. The obtained sequences of 16S rRNA gene fragments were then compared with the most closely related species available in the GenBank database using the NCBI Blast server. Additionally, the phylogenetic tree of bacterial isolates was constructed by MEGA X software using the method of maximum likelihood (ML) based on the 16S rDNA gene sequences.

## Results and Discussion

### Isolation and Molecular Identification of Bacteria From Qatari GTL Process Water

**Figure 2:**
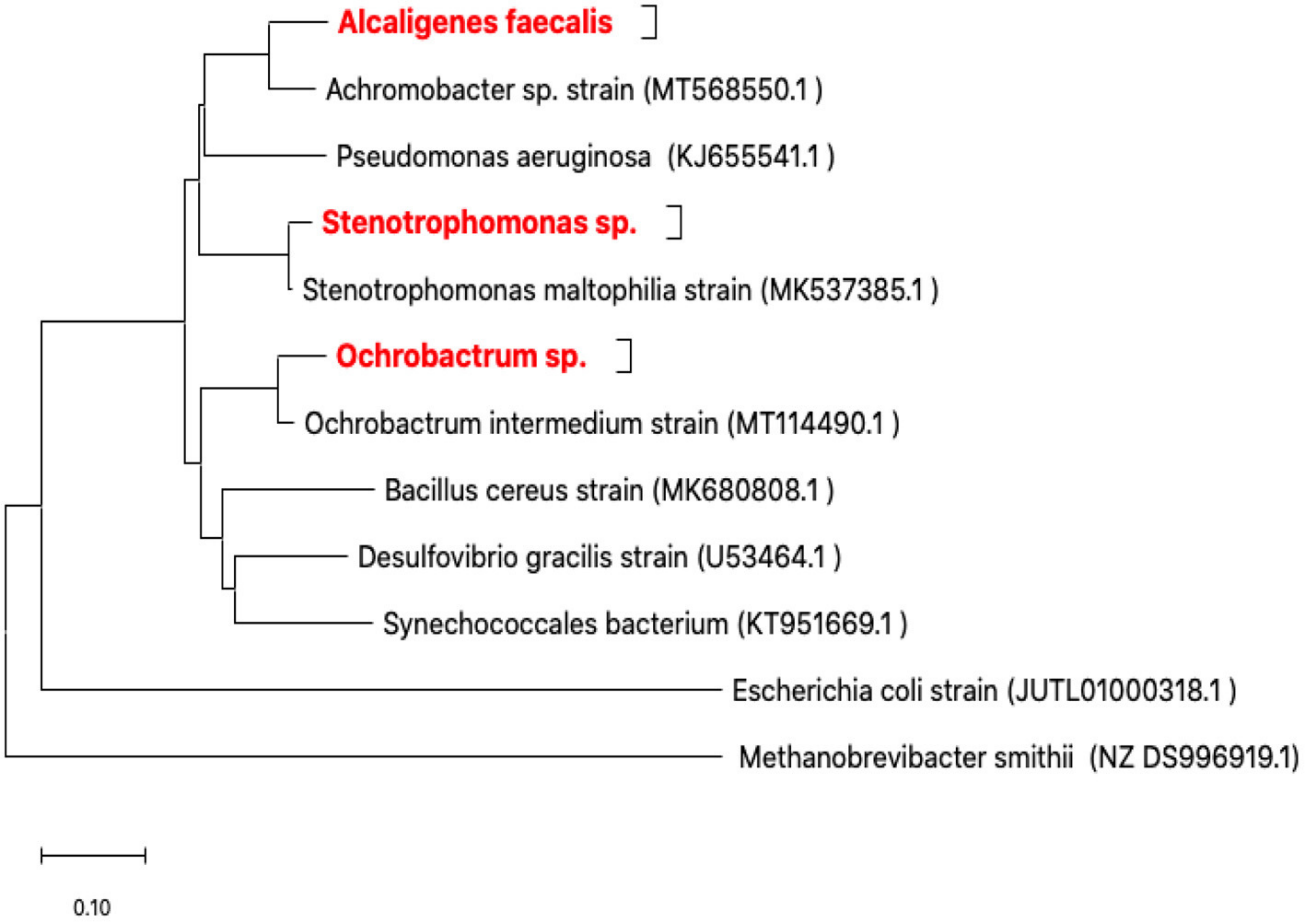
Neighbor-joining phylogenetic tree based on 16S rRNA gene sequences showing the positions of the studied strains. *Methanobrevibacter smithii* from Archaea was the domain used for outgroup rooting. *Achromobacter* sp., *Bacillus cereus, Desulfovibrio gracilis, Escherichia coli, Ochrobactrum intermedium, Pseudomonas aeruginosa, Stenotrophomonas maltophilia*, and *Synechococcales* bacterium were used as reference bacterial strains. GenBank accession numbers are given in parentheses. The evolutionary history was inferred using the neighbor-joining method (Saitou and Nei, 1987). The optimal tree with the sum of branch length = 2.51758258 is shown. The tree is drawn to scale, with branch lengths in the same units as those of the evolutionary distances used to infer the phylogenetic tree. The evolutionary distances were computed using the maximum composite likelihood method (Tamura et al., 2004) and are in the units of the number of base substitutions per site. This analysis involved 12 nucleotide sequences. All ambiguous positions were removed for each sequence pair (pairwise deletion option). There were a total of 1,790 positions in the final dataset. Evolutionary analyses were conducted in MEGA X (Kumar et al., 2018; Stecher et al., 2020).

Only three isolates (RZ3, RZ4, and RZ5) were obtained after enrichment cultures and isolation in LB medium. In fact, they represent three types of colonies characterized by their form and color and the microscopic observation of the corresponding bacteria. The isolates were identified by ribotyping, based on sequencing of their 16S rDNA amplicons, after purification. The obtained 16S rDNA sequence of each isolate was used to determine the most closely related sequence of available sequences in the GenBank database using the Blast server at NCBI. The three isolates identified were as follows: *Alcaligenes faecalis* (*RZ3*), *Stenotrophomonas* sp. (*RZ4*), and *Ochrobactrum* sp. (*RZ5*) (Figure 2).

It can be concluded that a few strains were isolated. There are two possible reasons for this. The water samples contained highly toxic organic compounds, so that only the three isolated strains were able to tolerate this high level of water toxicity. The enrichment medium contained low nutrient contents, which may not be enough to sustain the required nutrients for cell growth and maintenance. Therefore, a few cells would have been able to adapt and subsist. This result shows that the isolation strategy was efficient to bacteria by enriching cultures in LB containing GTL process water. This culture medium was very toxic to bacteria because of the high organic concentration (COD ranging from 6,000 to 7,000 mg/L). The aim of the isolation procedure was to isolate and purify bacteria with strong potentials to degrade and tolerate GTL process water.

The isolated bacteria have been widely applied in the treatment of different GTL process water containing a variety of organic contaminants. Table 2 summarizes the application of these strains in wastewater treatment in previous studies. *A. faecalis* shows high performance in the removal of ammonia from wastewaters (Neerackal et al., 2016). However, it has high tolerance in phenol removal from wastewater, even at very high initial concentrations (Bai et al., 2007). It was confirmed by Essam et al. (2010) that an isolated strain of *A. faecalis* has the ability to grow in phenol; however, it has difficulty to grow and remove other phenol derivatives, such as nitrophenols and chlorophenols. Additionally, the bacterium was shown with low tolerance to grow in alcoholic compounds such as methanol and ethanol (Essam et al., 2010). *Stenotrophomonas* sp. is reported with its tolerance in heavy metal removal (Gunasundari and Muthukumar, 2013); however, it showed good performance in the removal of phenols and organic carbon present in refinery wastewaters (Patel and Patel, 2020). *Ochrobactrum* sp. is a Gram-negative, rod-shaped, aerobic and oxidase-positive bacterium (Arulazhagan and Vasudevan, 2011). This bacterium showed high treatment performance of several contaminants present in wastewaters (Qiu et al., 2007). Neoh et al. (2016) showed that *Ochrobactrum* sp. has high performance in the biotreatment of agricultural wastewaters that contain high COD and nitrogen contents. In addition, *Ochrobactrum* sp. has high performance in the removal of phenol and its derivatives (El-Sayed et al., 2003; Qiu et al., 2007). The removal of the organic compounds present in the GTL process water was not documented using the isolated strains.

**Table 2:** List of previous works on the removal of organic compounds by the studied strains.

**Bacterial type**	**Bacterial source**	**Wastewater**	**Contaminant**	**Initial concentration (mg/L)**	**Removal %**	**Reference**
*Alcaligenes faecalis*	Durgapur steel industry wastewater	Synthetic	Phenol	1,600	100	Jiang et al., 2007
*Alcaligenes faecalis*	Activated sludge	Synthetic	Phenol	1,600	100	Jia et al., 2005
*Alcaligenes faecalis*	Activated sludge from coke factory	Synthetic	Phenol	1,000	100	Essam et al., 2010
*Stenotrophomonas* sp.	Polluted river	Petroleum wastewater	Phenols	998	89	Patel and Patel, 2020
*Stenotrophomonas* sp.	Polluted river	Petroleum wastewater	COD	15	93	Patel and Patel, 2020
*Stenotrophomonas* sp.	Aquifer	Synthetic	*p*-Nitrophenol	1.078	100	Subashchandrabose et al., 2013
*Ochrobactrum* sp.	Textile sludge	Agricultural	COD	11,707	71	Neoh et al., 2016
*Ochrobactrum* sp.	Textile sludge	Agricultural	Ammonium nitrogen	256	60	Neoh et al., 2016
*Ochrobactrum* sp.	Textile sludge	Agricultural	Total polyphenolic compounds	916	55	Neoh et al., 2016
*Ochrobactrum* sp.	Marine environment	Petroleum	PAHs, COD	1,000	66	Arulazhagan and Vasudevan, 2011
*Ochrobactrum* sp.	Phenol-activated sludge	–	Phenol	100	100	El-Sayed et al., 2003
*Ochrobactrum* sp.	Polluted soil	–	*p*-Nitrophenol (PNP)	100	100	Qiu et al., 2007

### Biodegradation of Organic Pollutants From GTL Process Water

#### Organic Removal Using Several Strains

**Figure 3:**
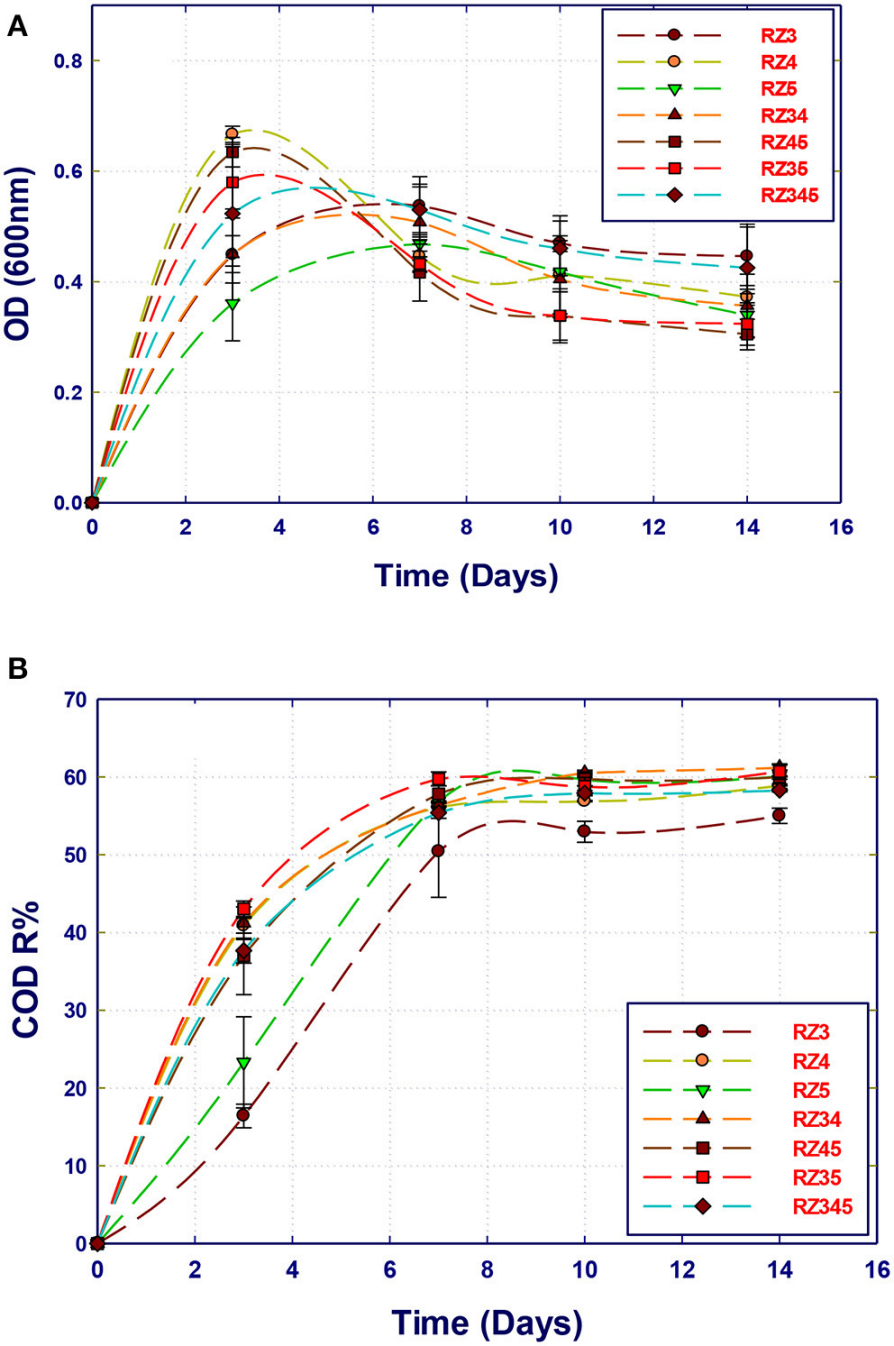
Comparison of the biotreatment of GTL process water using several bacterial strains: single, co-culture, and mixed culture. **(A)** Biomass growth; **(B)** COD reduction.

The potential of the isolated bacterial strains in the removal of organic compounds present in the Qatari GTL process water using pure and mixed cultures was evaluated. The GTL process water used in this section was obtained after the pretreatment process and has COD content of 1,800 mg/L. A set of 14 experiments were performed using the MSM-GTL medium to screen the isolated strains for the biodegradation of organic pollutants in GTL wastewater. All samples were kept in the incubator in which the COD content and biomass growth (biomass production) were evaluated at 3, 7, 10, and 14 days. The growth curves of single and mixed cultures are shown in Figure 2. It is clear that the three strains reach their maximum growth after 3 to 4 incubation days. Interestingly, the growth started immediately after inoculation, without a clear lag phase. This means that all the strains are highly adapted to the substrates, which are the organic compounds in GTL process water including alcohol, ketones, esters, and aliphatics. However, a clear decline in growth was observed after this period. The period of high growth was marked with a fast decrease of the COD content, which continued up to the 7th day (Figure 3). Nevertheless, there was no further COD reduction observed after a 1-week incubation. When comparing the growth curves and corresponding COD reduction curves for each single or mixed culture, it can be noticed that the strain *Stenotrophomonas* sp. (RZ4) showed the highest growth rate as a single culture, followed by the mixture *Stenotrophomonas* sp. with *Ochrobactrum* sp. (RZ5). Although *Ochrobactrum* sp. growth was the least as a single culture, a clear symbiotic and cooperation can improve the growth of *Ochrobactrum* sp. by the concomitant growth of *Stenotrophomonas* sp. A pure culture of *Ochrobactrum* sp. can also achieve around 56% COD reduction similar to that obtained by *Stenotrophomonas* sp. alone or by *Stenotrophomonas* sp. and *Ochrobactrum* sp. after 14 incubation days. However, the yield of *Ochrobactrum* sp. in biomass production was lower than that of *Stenotrophomonas* sp.

The strain *A. faecalis* (RZ3) also showed less growth rate than that of *Stenotrophomonas* sp. alone, which is reflected in a slightly lower COD reduction (52%). Mixing *A. faecalis* with *Stenotrophomonas* sp. did not improve its growth, although the COD reduction was improved to the best level. In contrast, the combination of the three strains resulted in biomass growth and COD reduction similar to that of *Stenotrophomonas* sp. alone. It seems that the strain *Stenotrophomonas* sp. can be used alone to remediate the Qatari GTL process water at these conditions (COD 1,800 mg/L).

### Effect of COD Concentration on Biodegradation Performance

**Figure 4:**
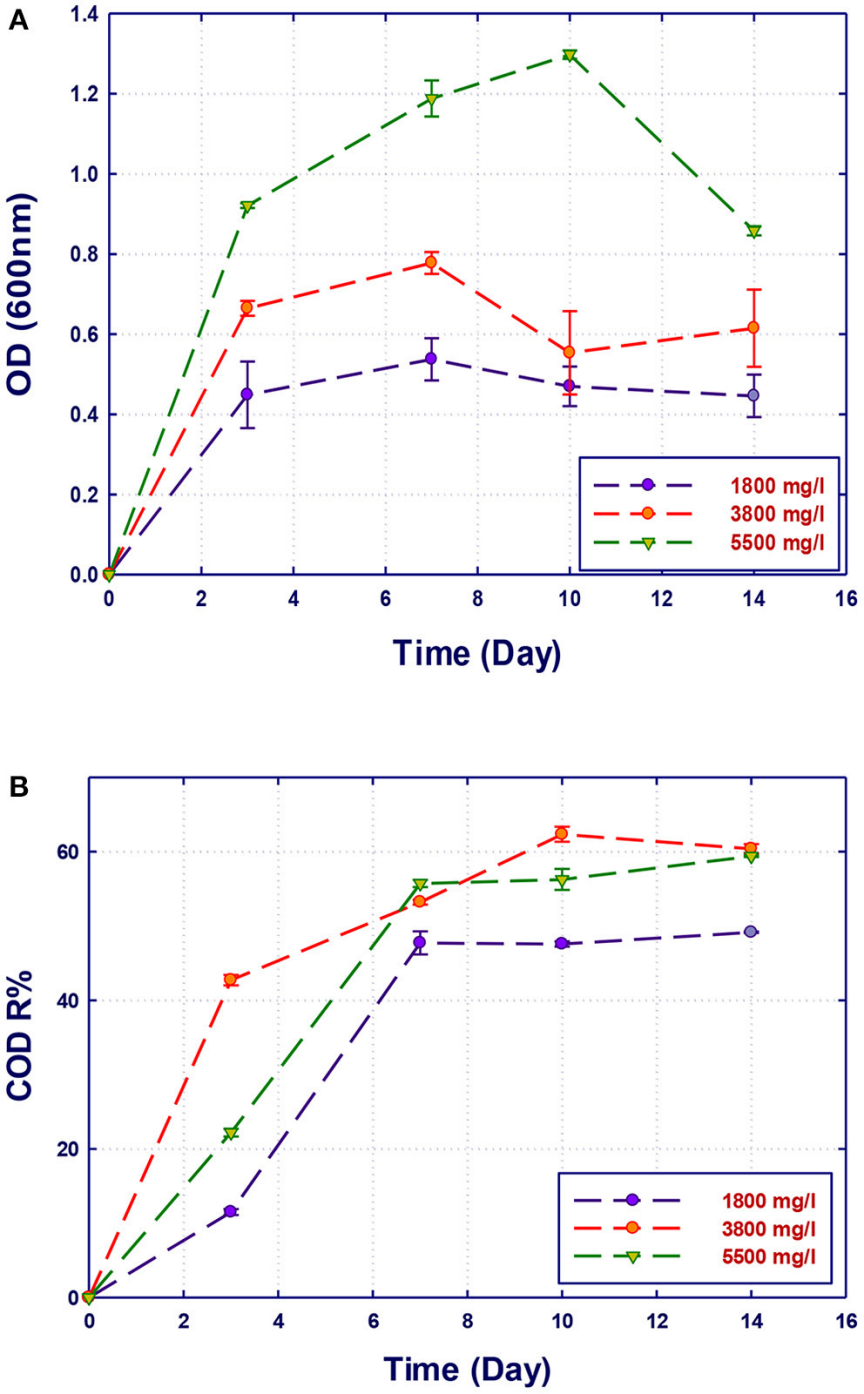
Biological treatment of GTL process water using *Alcaligenes faecalis* (RZ3). **(A)** Biomass growth; **(B)** COD reduction.

**Figure 5:**
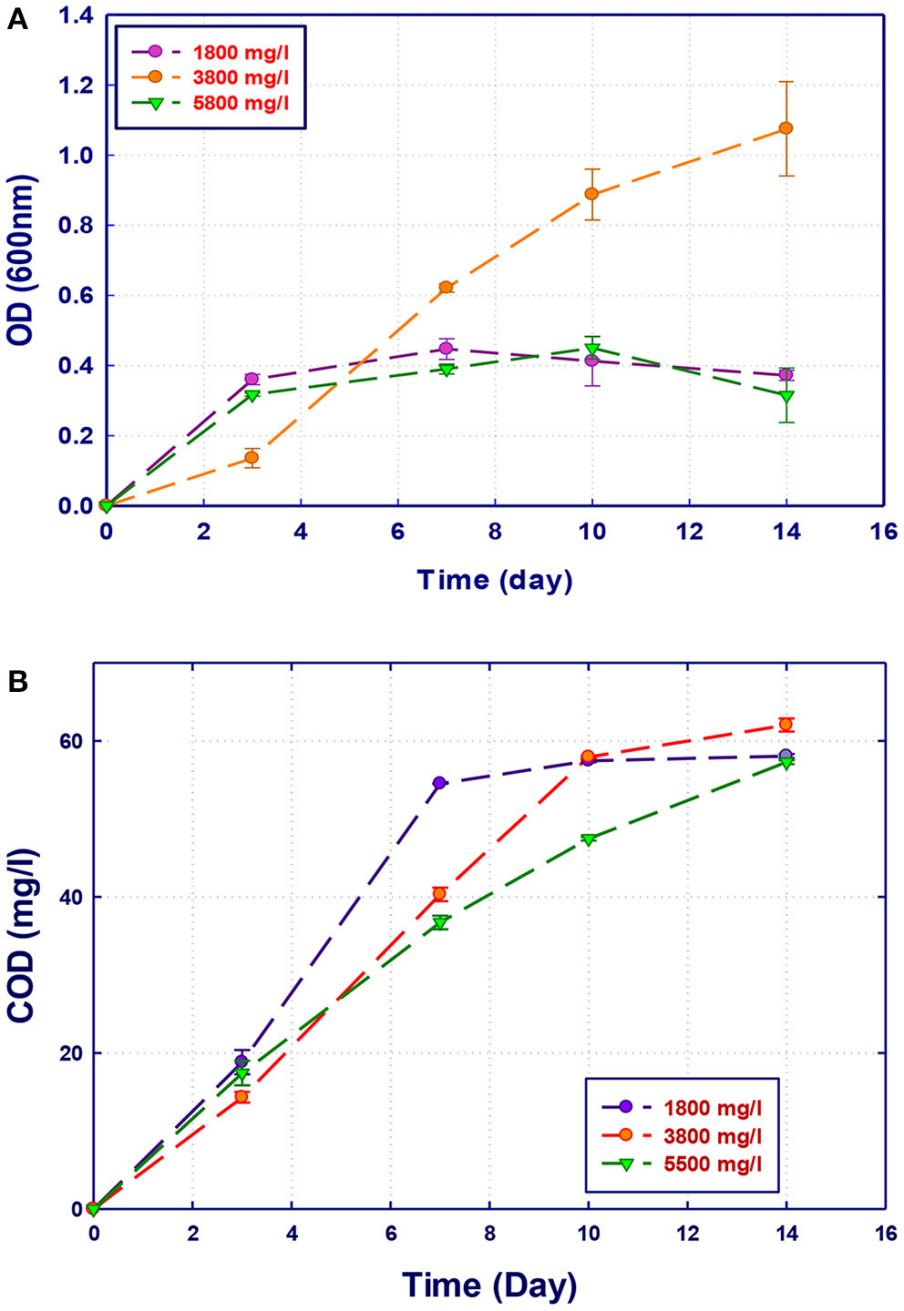
Biological treatment of GTL process water using *Stenotrophomonas* sp. (RZ4). **(A)** Biomass growth; **(B)** COD reduction.

**Figure 6:**
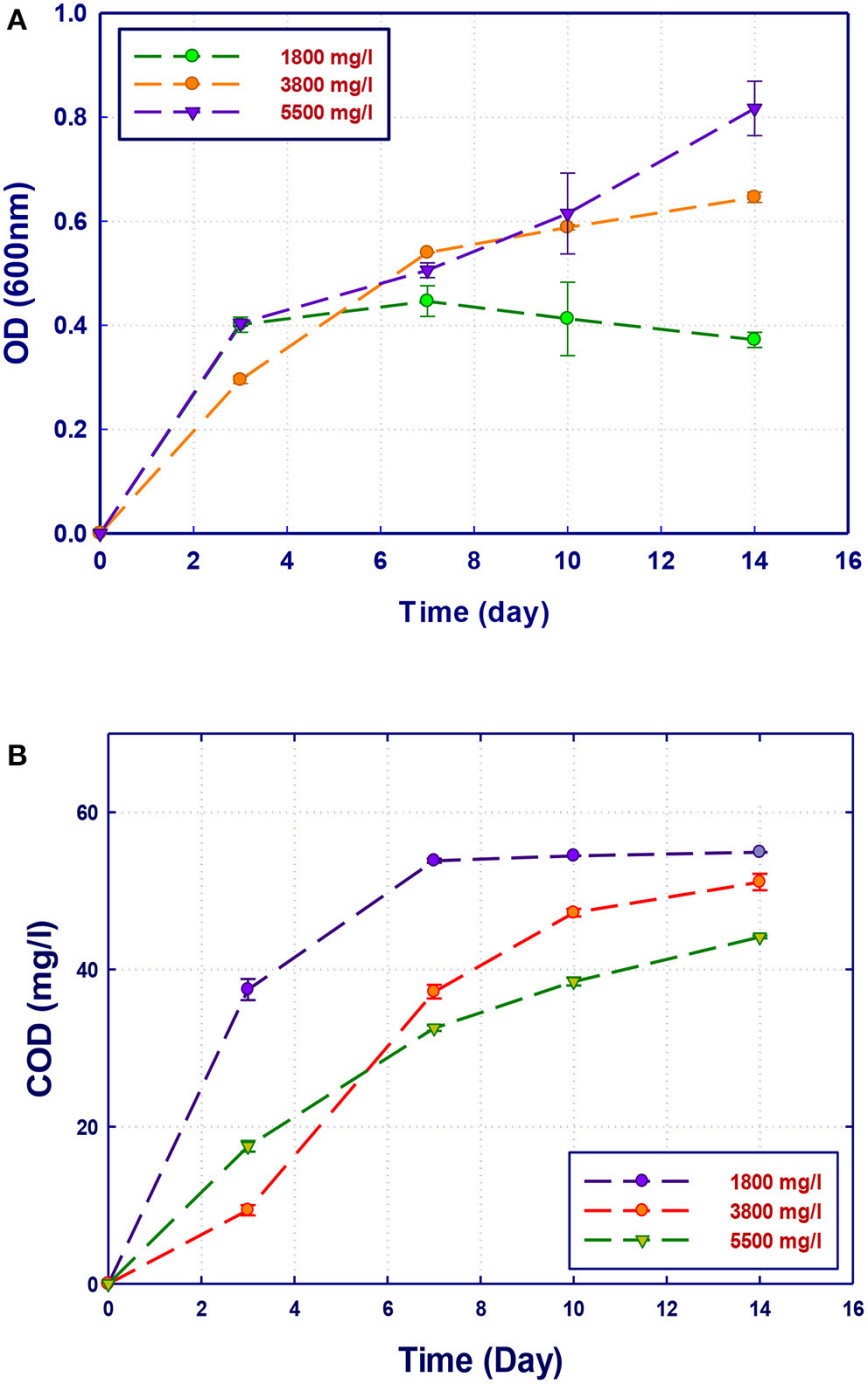
Biological treatment of GTL process water using *Ochrobactrum* sp. (RZ5). **(A)** Biomass growth; **(B)** COD reduction.

**Figure 7:**
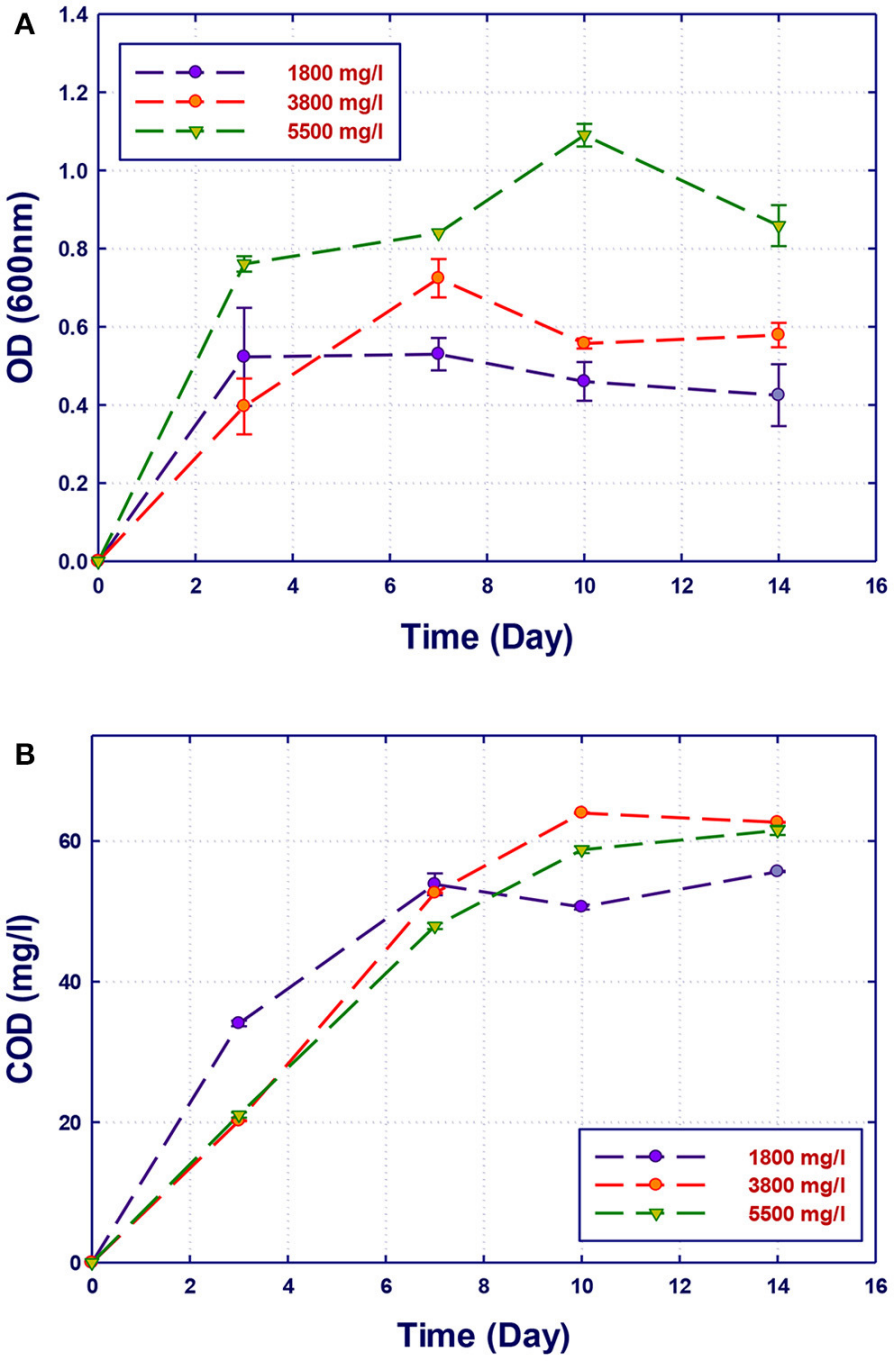
Biological treatment using mixed culture of *Alcaligenes faecalis, Stenotrophomonas* sp., and *Ochrobactrum* sp. (RZ 345). **(A)** Biomass growth; **(B)** COD reduction.

The performance of the isolated strains to grow and remove organics in GTL process water at three initial COD contents (1,800, 3,800, and 5,500 mg/L) was investigated. The initial COD content of the raw GTL process water was 5,500 mg/L, which was pretreated to reduce its concentration to 1,800 mg/L. The third COD content (3,800 mg/L) was obtained by diluting the initial raw GTL process water. The biodegradation performance of pure and mixed culture of the three strains was evaluated at each COD content. The biomass growth and COD reduction for each water sample (with different COD concentrations) are shown in Figures 4–7. Interestingly, the results of Figure 4 show that the strain *A. faecalis* (RZ3) is not inhibited by the excess of organic pollutants (high COD process water) since an increase of the growth rate is related to COD increase. Moreover, the overall removal of COD after 14 days of incubation was almost similar (60%). This may be attributed to the ability of *A. faecalis* to remove several organic pollutants in GTL wastewater including fatty acids that are available in the raw GTL process water.

The growth of both strains *Stenotrophomonas* sp. (RZ4) and *Ochrobactrum* sp. (RZ5) was negatively affected by the increase of the COD content in the growth medium. This means that these strains may be inhibited by the excess of substrates or inhibited by several organics present in GTL process water. The combination of the three strains showed high ability in the degradation of organic pollutants present in GTL process water, which indicates the synergy effect for several strains in the GTL wastewater treatment.

Although the three isolates were capable of degrading the organic pollutants in GTL process water at various COD contents, the removal of these organic contaminants by mixed cultures was improved compared with individual strains as shown in Figure 7. Each individual microorganism may have the ability to metabolize limited types of substrates; thus, the combination of different bacterial strains with wider enzymatic capabilities will result in the degradation of more organic pollutants giving higher COD reduction. This was confirmed by Senthilvelan et al. (2014) when they tested phenol degradation using single strains and mixed culture. In their study, a significant increase in the biodegradation rate and phenol removal was obtained using mixed microbial culture.

**Figure 8:**
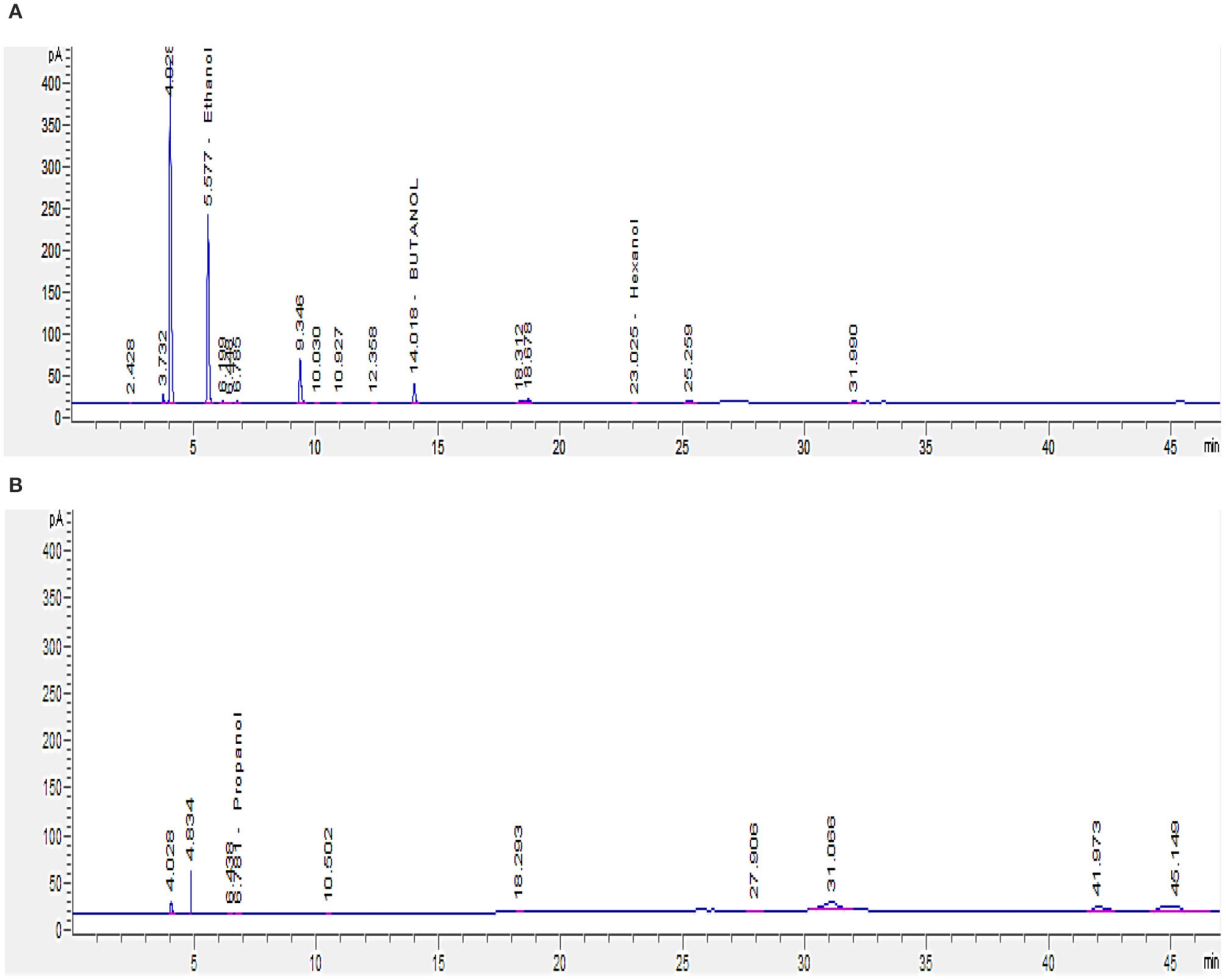
GC analysis for short-chain alcohols (C1–C6) in GTL process water: **(A)** before and **(B)** after biotreatment.

Since short-chain alcohols (SCA) represent about 82% of the COD content of GTL process water (Majone et al., 2010), GC analysis was carried out to determine their concentrations in GTL process water before and after biotreatment. GTL process water and biotreated water contain SCA (C1–C6) concentration of 2,465 and 813 mg/L, respectively (Figures 8A,B). The analysis showed that the isolated bacteria were able to degrade around 60% of the short-chain alcohols.

Since the SCA are the major contributor to the COD content in GTL process water, it is important to reduce their concentration through biotreatment, in order for the treated water to be reused or discharged safely.

It is obvious that GTL process water has a broader range of organic pollutants. This may require the application of several types of microorganisms in order to achieve high degradation performance. Thus, it is recommended to use mixed bacterial strains for the biological treatment of GTL process water, at different ranges of COD contents. The main novelty of this study is the application of the isolated strains to remove the organic pollutants in the same GTL process water and achieve high COD reduction under stress conditions, which has not been reported before.

## Conclusions

It is evident from this study that GTL process water is a rich source of organic components that are rather difficult to remove or degrade. However, the isolation, identification, and testing of organics-degrading bacterial strains, such as *A. faecalis, Stenotrophomonas* sp., and *Ochrobactrum* sp., from GTL water showed that these strains have the capability to degrade the organic contaminants in GTL water and can be a good alternative for the conventional activated sludge systems, which are currently applied in the biological treatment of GTL process water. Each bacterial strain has the ability to work as a single strain or in a mixed culture to remove the organic pollutants present in GTL process water. Regardless of the complex composition of GTL process water, the isolated strains resulted in high COD reduction (up to 60%) under stressed conditions and, consequently, had high growth performance in such industrial wastewater.

## Data Availability Statement

The datasets generated for this study can be found in online repositories. The names of the repository/repositories and accession number(s) can be found in the article/supplementary material.

## Author Contributions

RS, ME-N, and ZA conceived the original idea and designed the experimental program. RS and ZA carried out the experiments and prepared the initial draft. ME-N and NZ revised the manuscript with support from ZA and RS. MV and UO helped with discussion of the results and manuscript revisions.

## Conflict of Interest

The authors declare that the research was conducted in the absence of any commercial or financial relationships that could be construed as a potential conflict of interest.
